# Large Plasmid Complement Resolved: Complete Genome Sequencing of *Lactobacillus plantarum* MF1298, a Candidate Probiotic Strain Associated with Unfavorable Effect

**DOI:** 10.3390/microorganisms7080262

**Published:** 2019-08-14

**Authors:** Anette McLeod, Annette Fagerlund, Ida Rud, Lars Axelsson

**Affiliations:** 1 Nofima—Norwegian Institute of Food, Fisheries and Aquaculture Research, P.O. Box 210, N-1431 Ås, Norway; 2 Center for Laboratory Medicine, Østfold Hospital Trust, P.O. Box 300, N-1714 Grålum, Norway

**Keywords:** *Lactobacillus plantarum*, lactic acid bacterium, probiotic, PacBio sequencing, Illumina sequencing, Oxford Nanopore MinION sequencing, plasmid assembly

## Abstract

Considerable attention has been given to the species *Lactobacillus plantarum* regarding its probiotic potential. *L. plantarum* strains have shown health benefits in several studies, and even nonstrain-specific claims are allowed in certain markets. *L. plantarum* strain MF1298 was considered a candidate probiotic, demonstrating in vitro probiotic properties and the ability to survive passage through the human intestinal tract. However, the strain showed an unfavorable effect on symptoms in subjects with irritable bowel syndrome in a clinical trial. The properties and the genome of this strain are thus of general interest. Obtaining the complete genome of strain MF1298 proved difficult due to its large plasmid complement. Here, we exploit a combination of sequencing approaches to obtain the complete chromosome and plasmid assemblies of MF1298. The Oxford Nanopore Technologies MinION long-read sequencer was particularly useful in resolving the unusually large number of plasmids in the strain, 14 in total. The complete genome sequence of 3,576,440 basepairs contains 3272 protein-encoding genes, of which 315 are located on plasmids. Few unique regions were found in comparison with other *L. plantarum* genomes. Notably, however, one of the plasmids contains genes related to vitamin B12 (cobalamin) turnover and genes encoding bacterial reverse transcriptases, features not previously reported for *L. plantarum*. The extensive plasmid information will be important for future studies with this strain.

## 1. Introduction

*Lactobacillus plantarum* is one of the most versatile species among lactic acid bacteria (LAB) [1]. Strains of the species are able to colonize a variety of environments including vegetables, meat, dairy substrates and the gastrointestinal (GI) tract [2,3]. There has been considerable interest in the probiotic potential of *L. plantarum* to maintain and regulate the human intestinal microbiota [4,5], and health benefits have been presented [3,6,7]. *L. plantarum* belongs to a list of species that has been suggested to be of "general benefit", and for which nonstrain-specific health claims can be made in certain markets [8]. The largest successful clinical trial to date of an oral probiotic preparation was recently reported by Panigrahi et al. [9]. Their findings suggest that a large proportion of neonatal sepsis in developing countries could be effectively prevented using a synbiotic containing *L. plantarum* ATCC 202195. Although most commercially available probiotic strains are widely regarded as safe, concerns have been raised. Initially, these concerns were mainly with respect to safety in particular populations [10,11]. Recently, concerns of a more general nature have also been put forward, especially with regard to the effects on the microbiome equilibrium in the GI tract [12]. Published reports of unfavorable effects of probiotics are scarce [12]. *L. plantarum* strain MF1298, originating from a single colony from Norwegian mutton salami [13], showed promising probiotic potential in early investigations. The strain was confirmed to have antimicrobial activity against potential pathogens, to adhere to the human colon adenoma cell line CaCo-2, and to strengthen transepithelial resistance of a CaCo-2 cell layer with increased production of tight junction proteins [13,14]. Tight junctions consist of protein complexes that maintain epithelial barrier integrity, providing an important primary barrier for the intestinal space. The strain was also shown to survive passage through the human GI tract [15]. Despite these suitable properties, an unfavorable effect on symptoms in subjects with irritable bowel syndrome (IBS) was shown after intake of *L. plantarum* MF1298 in a randomized placebo-controlled crossover trial [16,17]. This unfavorable effect manifested itself as a slight, but statistically significant, increase in the standard IBS score. The score is based on a patient’s subjective experience of GI function during a treatment compared to a control period, such as “abdominal pain/discomfort”, “urgency” and “stool frequency/consistency” [17]. For this reason, the properties and the genome sequence of this strain are interesting, as they may contribute to a broader understanding of probiotics in general and the inspection of genome sequences of candidate probiotic strains with regard to safety and potential undesirable effects.

Whole genome sequencing (WGS) has revolutionized the characterization of bacteria. The sequence of a bacterial genome can be obtained in a very short timeframe and at low costs. However, there are still challenges with regard to obtaining a complete and correctly assembled genome. Bacteria generally harbor a large circular chromosome, and different strains may, in addition, contain one or multiple extrachromosomal plasmids with varying sizes and copy numbers. Plasmids present important genetic engineering tools and are transmission vectors of genes that in nature may benefit the survival of the organism, including resistance to antibiotics, bacteriophages, toxic heavy metals and other stress responses, the production of bacteriocin and exopolysaccharide, and the provision of enzymes that expand the nutritional ability of the cell [18]. The application of short-read sequencing technologies, such as Illumina, to provide WGS data tends to generate incomplete, fragmented genome assemblies, in which differentiation between plasmid and chromosome sequences may be impossible. The correct assembly of the plasmid fraction using short reads is hindered by the presence of high levels of repetitive DNA that is shared between several plasmids and the chromosome [19]. Bacterial plasmids can also span hundreds of kilobases (kb), adding another level of complexity to accurate assembly. Long-read sequencing technologies developed by Pacific Biosciences (PacBio) and Oxford Nanopore Technologies (ONT) overcome many of the limitations researchers face with short reads [20]. However, while standard long-read sequencing protocols often employed in PacBio sequencing include a size selection step that may exclude smaller plasmids [19], nanopore sequencing using ONT platforms has been employed to decipher the structures of smaller plasmids [21,22]. A unique property of the ONT MinION platform is its size, cost, portability, and the real-time utility, where very long reads are rapidly generated [23].

Here, the complete genome sequence of *L. plantarum* MF1298 is reported, which consists of one circular chromosome and an unusually high number of plasmids: 14 circular plasmids ranging in size from 2.3 to 63.1 kb were identified. The accurate complete sequence of MF1298 was obtained in a stepwise approach, in which long-read PacBio sequencing was initially used to obtain the sequence of the chromosome and the two largest plasmids. Subsequent short-read Illumina sequencing of the total genomic DNA fraction showed that a substantial amount of plasmid content was lacking from the PacBio assembly. To resolve the plasmid complement, known to often contain genes associated with important functions in LAB strains [18], the plasmid DNA fraction was sequenced using both Illumina and long-read ONT MinION sequencing, and a hybrid assembly was generated using the combined data.

## 2. Materials and Methods

### 2.1. Growth Conditions and DNA Preparation

*L. plantarum* MF1298 [13] was cultivated in rich MRS broth (Oxoid, Thermo Fisher Microbiology, Basingstoke, UK) at 37 °C overnight (still culture). Total genomic DNA was extracted with Advamax beads (Edge BioSystems, Gaithersburg, MD, USA) as detailed elsewhere [24]. The plasmid DNA fraction was purified using the Qiagen Large-Construct Kit (Qiagen, Hilden, Germany)). An additional lysis step was introduced where the cells were incubated at 37 °C for 10 min in lysis buffer-added lysozyme (20 mg/mL) (Sigma Aldrich, Steinheim am Albuch, Germany) and mutanolysin (40 U/mL) (Sigma Aldrich). With the plasmid DNA preparation, a sufficient depth of coverage for all plasmids was more likely to be reached, and this strategy therefore avoids omissions from final assembly. The DNA quality was assessed by 0.8% agarose gel electrophoresis; concentration and purity (A260/A280) were measured using NanoDrop ND-1000 (Thermo Fisher Scientific, Waltham, MA, USA) and Qubit 3 Fluorometer (Thermo Fisher Scientific). DNA samples were preserved at −20 °C until further processing.

### 2.2. Genome Sequencing and Assembly

The total genomic DNA preparation was sequenced with PacBio RSII (Pacific Bioscience, Menlo Park, CA, USA) and Illumina MiSeq (San Diego, CA, USA). The RSII library was constructed using the 10 kb-protocol with BluePippin (Sage Science, Beverly, MA, USA) size selection, and sequences were generated using P4-C2 chemistry and two single-molecule real-time (SMRT) cells. An Illumina Nextera XT library was prepared according to the manufacturer’s protocols and sequenced using the MiSeq instrument with 300-bp paired-end reads. In total, 58,524 PacBio reads with an average length of 7893 bp were obtained, generating a total number of 462 Mbp. The raw reads were filtered prior to de novo assembly using HGAP v2 (Pacific Bioscience). This assembly generated five PacBio contigs, one large (>3 Mbp) and four smaller (<100 Kbp). The two smallest contigs had an average coverage of <30, and were excluded from further analysis. The three larger contigs had an average coverage of >50 and obvious self-overlapping regions at the beginning and end. Illumina MiSeq sequencing resulted in a total of approximately 13,000,000 good quality pair-end reads, which were used for error correction and confirmation of circularization of the three PacBio contigs using CLC Genomics Workbench v.6.0 (Qiagen). Subsequently, a separate assembly was made from the Illumina reads using CLC Genomics Workbench. The Illumina assembly gave 135 contigs of >500 bp, excluding contigs with coverages of <100×. The average coverage of contigs mapping to the largest PacBio contig was approximately 1400×.

The plasmid DNA preparation was sequenced by Illumina MiSeq, a Nextera XT library was prepared and sequenced as described above and also by ONT MinION (Oxford Nanopore Technologies, Oxford, UK) sequencing. Two runs were performed on the ONT MinION sequencer using R9.4/FLO-MIN106 flow cells. Sequencing libraries were prepared using the ligation sequencing kit 1D (SQK-LSK108), following the manufacturer’s protocols. In the first run, the DNA was sheared by passing through a 21G needle 20 times, and the library was barcoded using the Native barcoding kit (EXP-NBD103) (Oxford Nanopore Technologies). In the second run, the DNA was sheared to approximately 8000-kb fragments in a g-TUBE (Covaris, Brighton, UK). Raw nanopore fast5 reads were base-called using ONT-Albacore v.2.0.2 (Oxford Nanopore Technologies), and adapters were removed using Porechop v.0.2.2 (Oxford Nanopore Technologies). For the reads from the barcoded library, both ONT-Albacore and Porechop were run with barcode demultiplexing. A total of 954,040 good quality pair-end Illumina reads and 26,690 nanopore reads >1 kb (mean read length of 5880 bp) were assembled using the Unicycler v.0.3.0b hybrid assembly pipeline [25], resulting in 12 distinct closed circular plasmids.

### 2.3. Genomic Analysis

Genomic features were identified and annotated using the NCBI Prokaryotic Annotation Pipeline (PGAP) [26]. Chromosome alignments between MF1298 and five other *L. plantarum* strains were performed using the progressive MAUVE algorithm of the MAUVE software [27] with default options. The strains used were (GenBank no.): *L. plantarum* WCFS1 (AL935263.2), *L. plantarum* TMW 1.1623 (CP017379.1), *L. plantarum* ZJ316 (CP004082.1), *L. plantarum* KP (CP013749.1) and *L. plantarum* subsp. *argentoratensis* DSM 16365^T^ (CP032751.1). The chromosome DNA sequences were arranged to have the same orientation and start position (gene *dnaA*) before alignment.

Plasmids and selected chromosomal sequence regions were subjected to homology searches using BLAST [28]. In some instances, genomic features were identified and annotated using the Rapid Annotation Search Tool (RAST) [29] to complement the PGAP annotation.

## 3. Results and Discussion

### 3.1. Genome Characteristics

The initial total genomic DNA sequencing with PacBio and Illumina MiSeq strategies yielded three circular units, one large circular chromosome of 3,235,952 bp and two plasmids of 63,114 bp and 55,699 bp (Table 1). The first version of the genome assembly (GenBank no. GCA_001880185.1) also contained 26 linear contigs originating from a separate Illumina assembly, in which contigs mapping to the three PacBio units were excluded, and a contig-size cutoff of 1000 bp was used. The total length of the 26 contigs was 209,814 bp, showing that a considerable part of the plasmid DNA was not captured or assembled correctly using the PacBio approach. The Illumina assembly alone was, however, too fragmented to resolve these sequences into circular units. To resolve the plasmid fraction, we therefore applied a hybrid approach with Illumina MiSeq and ONT MinION sequencing of a plasmid DNA-enriched fraction. Indeed, this hybrid assembly of nanopore long-reads and Illumina short-reads yielded 12 circular plasmids that ranged in size from 2273 to 47,476 bp (Table 1).

The large plasmids obtained from PacBio sequencing were poorly represented in this plasmid fraction, possibly reflecting the generally recognized problem of recovering large plasmids with standard plasmid enrichment procedures [30]. General genome features are presented in Table 1 and approximate read coverages listed in Table S1. The size of the complete *L. plantarum* MF1298 genome was 3,576,440 bp in total. This is one of the largest complete genomes described so far for *L. plantarum*, and the plasmid complement is the largest, both in the number of plasmids (14) and the bp content (340,488). Previously, *L. plantarum* strain 16 harbored the largest plasmid complement reported for this species, involving 10 plasmids (pLp16A–pLp16L), which ranged in size from 6.46 to 74.08 kb [31]. The GC content of the complete genome was 44.2%, with the chromosome specifically at 44.6%, similar to other *L. plantarum* strains. The plasmids generally had significantly lower GC content (Table 1), indicating that these might originate from horizontal gene transfer (HGT) events [32]. The plasmids contribute 315 protein-encoding genes to *L. plantarum* MF1298 and increase the total genomic content by 10.5%.

**Figure 1:**
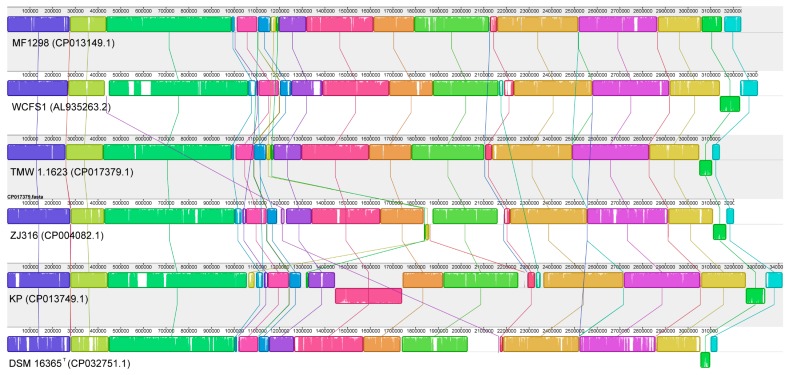
Chromosomal alignment of six *Lactobacillus plantarum* strains. The alignment was performed using MAUVE software [27] with strain MF1298 set as the reference. Each contiguously colored region is a local collinear block (LCB), a region without rearrangement of homologous backbone sequence. LCBs below the center line of a chromosome are in the reverse complement orientation relative to the reference chromosome. Lines between genomes trace each orthologous LCB through every chromosome. The white areas inside each LCB show regions with low similarities. Regions outside LCBs represent unique regions.

MAUVE alignments between the MF1298 chromosome and a large number of other complete *L. plantarum* chromosomes were performed, and a high degree of synteny could be seen between the chromosome of MF1298 and that of the other strains (not shown). We carefully selected five of the complete chromosomes of the subspecies *plantarum*, which included a standard reference strain (strain WCFS1 [33,34]), one of the largest *L. plantarum* chromosomes to date (the insect strain KP [35]), and two strains (TMW 1.1623 and ZJ316) that represent genomes at different distances from MF1298 in the phylogeny of *L. plantarum* genomes as presented by NCBI [36]. Finally, we also included the complete chromosome of a strain representing an example of the subspecies *argentoratensis* (DSM 16365^T^). The high degree of synteny between the chromosome of MF1298 and that of the other strains is clearly illustrated (Figure 1), as well as a high similarity within each local collinear block (LCB). MF1298 has an inversion compared to the other strains in the so-called Lifestyle Adaptation Region [33] near the end of the linear representation of the chromosome (position approximately 3,100,000), and contains a few unique regions in comparison with the five other strains. However, BLAST homology searches revealed that the genes present in these regions are found in other *L. plantarum* strains not included in this comparison (not shown), and are therefore not unique for the species as a whole. As observed previously [1], the larger unique regions found in all strains often represent different prophages. The chromosome from strain DSM 16365^T^, representing the subspecies *argentoratensis*, appeared most divergent from the others, which was expected and also noted by others [1].

### 3.2. The Plasmid Complement

**Figure 2:**
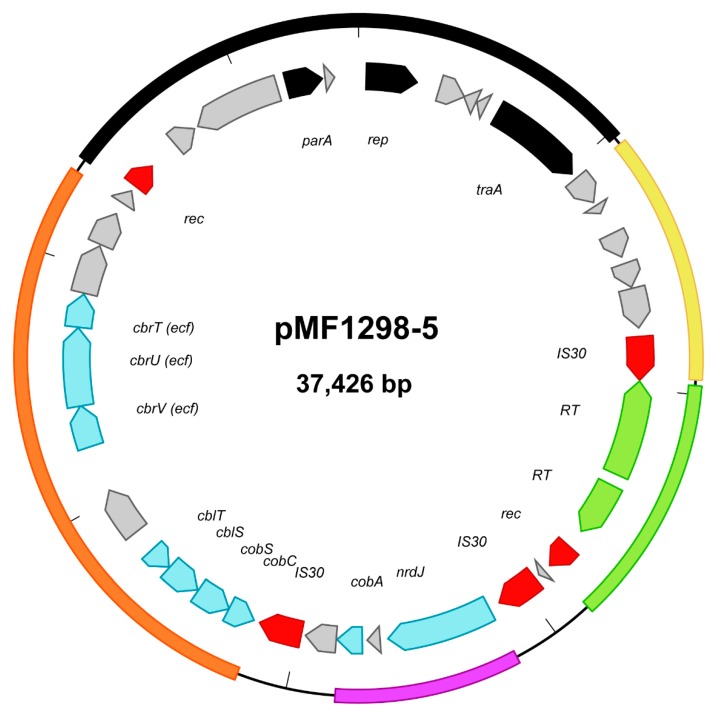
*Lactobacillus plantarum* plasmid pMF1298-5 displays a mosaic structure with regions of different origins flanked by transposases/recombinases: *L. plantarum rep/parA* (replication and partition) and *tra* (conjugal transfer) region (black), *L. plantarum* and *Pediococcus* plasmids (yellow), *Pediococcus parvulus* retron (reverse transcriptase; RT) region (green), *L. buchneri/L. brevis nrdJ/cobA* region (purple), *L. brevis* cobalamin region (orange). Genes related to cobalamin turnover are depicted in light blue and transposase/recombinase genes in red. Genes not annotated in this illustration are shown in grey (a majority of these encode hypothetical proteins). See text for further details. Annotations of the genes are based on RAST [29] and BLAST [28] homology searches in addition to the primary PGAP annotation (GenBank no. CP013155.2).

Plasmid maps of pMF1298-1 to -14 are presented in the Supplementary Materials (Figure S1). Inferred by homology using BLAST [28], the four smallest plasmids (pMF1298-11 to -14) appear to have a rolling-circle (RC) type of replication, with replication genes (*rep*) similar to published *L. plantarum* plasmids [37]. The other plasmids most likely have a theta type of replication, showing *rep*-regions with homology to either known *L. plantarum* plasmids [38] or to the same family as pAD1 or pAMβ1 archetypal conjugative enterococcal plasmids [39]. Accordingly, some of the larger plasmids contain genes encoding putative functions involved in conjugal transfer and mobilization. Putative mobilization genes were also represented in some of the smaller plasmids. Coverage data from the initial Illumina MiSeq assembly of total genomic DNA (Table S1) indicated copy numbers of 8–12 chromosome equivalents for the RC plasmids. The other plasmids have considerably lower copy numbers (<4). Note that the Illumina sequencing coverage data of the plasmid-enriched fraction showed the same, even more pronounced, division of high- and low-copy number plasmids (Table S1). In this case, plasmid enrichment may have introduced a bias towards higher recovery of the smaller, high-copy number plasmids. Most of the genes encoded by the MF1298 plasmids have homologues in other *L. plantarum* strains; however, the similarities are limited to relatively short stretches of homology. In many cases, contiguous stretches of homologous gene sequences are interrupted by transposable elements and/or recombinase genes (approximately 50) scattered among the plasmids (Figure S1). This creates composite or mosaic structures, which has been noticed for *L. plantarum* plasmids previously [38]. An example of this is shown in more detail for plasmid pMF1298-5 (Figure 2).

Thus, the picture that emerges is of a strain that has acquired a substantial amount of extrachromosomal elements through HGT, followed by extensive rearrangements mediated by an array of transposons/recombinases, and in addition may have the capability to act as a donor for new HGT events through conjugation and mobilization. This reinforces the notion that *L. plantarum* represents an example of a nomadic bacterial species that is characterized by its dynamic and flexible lifestyle [2], and where the plasmid biology may play an important, possibly underestimated, role. The sheer amount of plasmid DNA and the combination of genes thus acquired define some of the uniqueness of strain MF1298. In addition, and in contrast to the chromosome, plasmid pMF1298-5 (Figure 2) contains regions that appear to be unique for MF1298 compared to other *L. plantarum* strains. These regions contain genes putatively encoding functions related to vitamin B12 (cobalamin) turnover: a coenzyme B12 dependent ribonucleotide reductase (NrdJ), Cob(I)alamin adenosyltransferase (CobA), an operon encoding homologues to the proteins CblT, CblS, CobS, and CobC, which are related to a salvage pathway for cobalamin synthesis [40], and an ECF transporter (CbrV, CbrU, CbrT) [41]. Providing appropriate precursors are available, this may enable strain MF1298 to produce cobalamin. Interestingly, an adjacent region in this plasmid includes genes encoding two retron-type reverse transcriptases [42], also unique among *L. plantarum* strains, indicating the presence of yet another type of mechanism that might increase the flexibility of the genome. Also worthy of note, and due to its plasmid complement, strain MF1298 seems particularly well equipped for heavy-metal resistance. Two identical copies of the arsenate and cadmium resistance gene operon (*ars*/*cad*), known from a plasmid of the reference strain *L. plantarum* WCFS1 [43], are present on pMF1298-1 and pMF1298-2. In addition, other genes putatively related to heavy-metal resistance are present on pMF1298-4, pMF1298-6 and pMF1298-7 (Figure S1).

### 3.3. Probiotic Potential

Despite being shown to have suitable probiotic properties in vitro and to survive passage through the human intestinal tract [13,14,15], *L. plantarum* MF1298 was associated with an unfavorable effect on symptoms in subjects with IBS when tried as a candidate probiotic [16,17]. Our genome analysis supported some of the potential probiotic properties of the strain. For example, genes encoding large surface proteins or extracellular matrix-binding proteins, such as mucus- or collagen-binding proteins (e.g., protein_IDs APD01026.1, APD01681.1, APD01745.1, and AXN90884.1), which might enable bacterial attachment to eukaryotic epithelial cells, were identified. Some of these were plasmid-encoded. Although difficult to evaluate from genome data only, this initial investigation did not reveal obvious genome features that can explain the unfavorable effect found in the clinical trial.

Since the IBS diagnosis is based on subjective patient experience, such as “abdominal pain”, and not measurements of specific physiological parameters, speculations on which genes might be involved in creating aggravated symptoms become difficult. MF1298 did not show any unusual antibiotic resistance pattern in a simple phenotypic test performed prior to the clinical trial (unpublished observations). This was confirmed in this work as no antibiotic resistance genes were identified. Similarly, no virulence factors were found. Except for a version of the plantaricin operon in the chromosome, present in most *L. plantarum* strains [1,2], bacteriocin genes were also not identified.

## 4. Conclusions

In all, information on the whole genome sequence of *L. plantarum* MF1298 presented here will be useful for further studies of this strain for evaluating its properties in relation to genome content. Further investigations on strain MF1298 may also contribute to a deeper understanding of which genes and corresponding properties may define a probiotic and/or may constitute a possible safety concern. Considering the high genetic versatility of the *L. plantarum* species [1,2], it is valuable to increase the number of sequenced strains to account for the genetic variability and their association with specific features like probiotic potential. Such studies should include complete assemblies of the plasmid content since, as shown for strain MF1298, as much as 10% of the genome content, as well as unique features, could be located on plasmids.

## Figures and Tables

**Table 1:** Genome features of *Lactobacillus plantarum* MF1298.

GenBank Assembly *	GenBank No.	Type	Name	Topology	Size (bp)	GC%	Protein	rRNA	tRNA	Other RNA	Gene	Pseudo Gene
GCA_001880185.1 and GCA_001880185.2	CP013149.1	Chromosome	MF1298	Circular	3235952	44.6	2957	16	69	4	3118	72
	CP013150.1	Plasmid	pMF1298-1	Circular	63114	41.1	60	-	-	-	71	11
	CP013151.1	Plasmid	pMF1298-2	Circular	55699	40.1	50	-	-	-	58	8
GCA_001880185.2	CP013153.2	Plasmid	pMF1298-3	Circular	47476	40.7	40	-	-	-	47	7
	CP013152.2	Plasmid	pMF1298-4	Circular	45064	40.8	35	-	-	-	49	14
	CP013155.2	Plasmid	pMF1298-5	Circular	37426	40.7	33	-	-	-	38	5
	CP013156.2	Plasmid	pMF1298-6	Circular	30526	45.8	29	-	-	-	35	6
	CP013154.2	Plasmid	pMF1298-7	Circular	23493	39.6	21	-	-	-	29	8
	CP013158.2	Plasmid	pMF1298-8	Circular	10848	36.5	14	-	-	-	17	3
	CP013160.2	Plasmid	pMF1298-9	Circular	8511	34.2	10	-	-	-	10	-
	CP013166.2	Plasmid	pMF1298-10	Circular	5636	36.3	9	-	-	-	11	2
	CP013162.2	Plasmid	pMF1298-11	Circular	4130	37.7	4	-	-	-	4	-
	CP013167.2	Plasmid	pMF1298-12	Circular	3350	38.4	4	-	-	-	4	-
	CP013168.2	Plasmid	pMF1298-13	Circular	2942	35.8	3	-	-	-	5	2
	CP013170.2	Plasmid	pMF1298-14	Circular	2273	36.6	3	-	-	-	3	-

* The initial version 1 (GCA_001880185.1) of the genome assembly was based on PacBio and Illumina sequencing data of total genomic DNA and consisted of 29 entities. It included three circular units, one large chromosome and two plasmids, as well as 26 linear contigs (the linear contigs are not indicated in this table). The three circular units remain unchanged in the second version (Version 2; GCA_001880185.2), consisting of 15 entities, which included MinION and Illumina sequencing data of a plasmid DNA-enriched fraction, which facilitated the recovery of complete plasmid structures.

## Supplementary Materials

The following are available online at https://www.mdpi.com/2076-2607/7/8/262/s1, Table S1: Sequence coverage, Figure S1: *Lactobacillus plantarum* plasmids pMF1298-1 to -14.
