# Supplementary material for: Large Plasmid Complement Resolved: Complete Genome Sequencing of Lactobacillus plantarum MF1298, a Candidate Probiotic Strain Associated with Unfavorable Effect
Source: Microorganisms. 2019 Aug 14;7(8):262. doi: 10.3390/microorganisms7080262 (PMC6722938; doi:10.3390/microorganisms7080262)
Supplement: Supplementary file 1 [file microorganisms-07-00262-s001.zip › McLeod MF1298_Genome_revised_final_Figure S1.pdf]

Supplementary material – McLeod MF1298_Genome_final - revised

Figure S1 ǀ *Lactobacillus plantarum* MF1298 plasmids pMF1298-1 to -14.

The next 7 pages represent the full plasmid complement of strain MF1298 by plasmid map overviews with all genes depicted, including locus tags. For a full annotation, the reader is referred to the GenBank assembly accession: GCA_001880185.2 (NCBI Assembly: ASM188018v2; https://www.ncbi.nlm.nih.gov/assembly/GCF_001880185.2) and the corresponding GenBank entries (see Table 1 and Table S1).

Certain gene categories, some of which are mentioned in the main text of this article, are highlighted with colors:
1) transposons/recombinases (red); 2) replication/mobilization/conjugation (black); 3) heavy-metal resistance (brown); 4) toxin-antitoxin systems (plasmid maintenance) (yellow); 5) cobalamin turnover (blue); 6) retron-type reverse transcriptases (green); 7) extra-cellular matrix binding protein (purple).

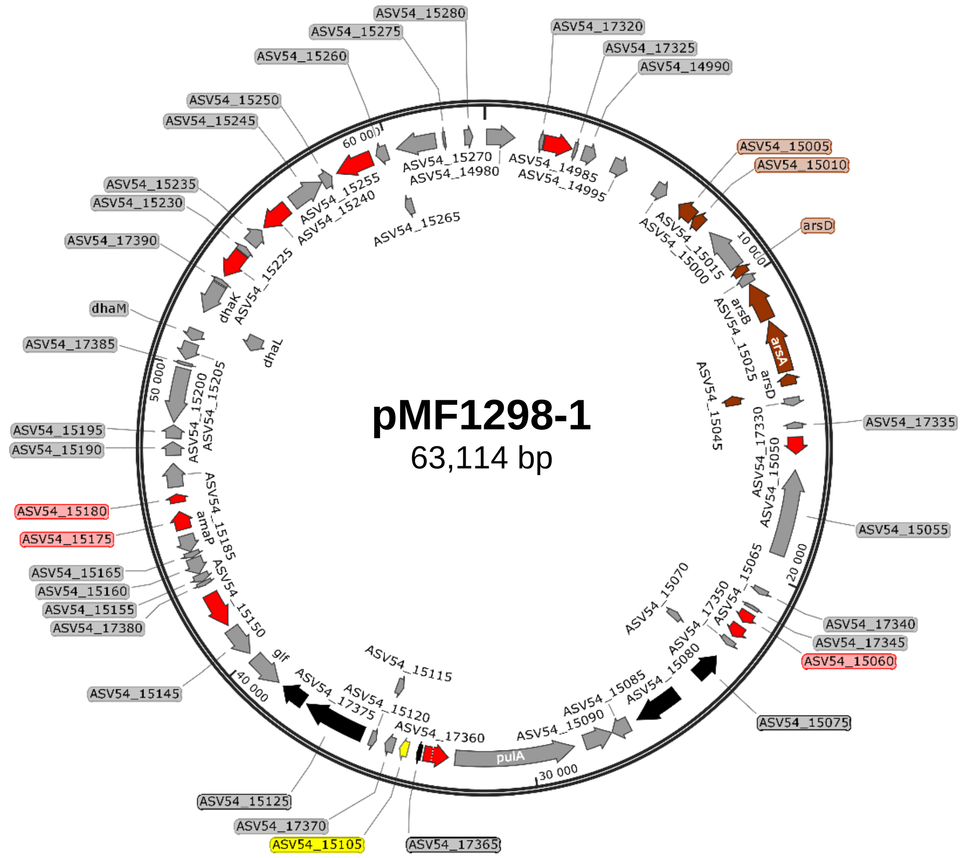
pMF1298-1
63,114 bp
ASV54_15280
ASV54_15275
ASV54_15260
ASV54_15250
ASV54_15245
ASV54_15235
ASV54_15230
ASV54_17390
dhaM
ASV54_17385
ASV54_15195
ASV54_15190
ASV54_15180
ASV54_15175
ASV54_15165
ASV54_15160
ASV54_15155
ASV54_17380
ASV54_15145
ASV54_15125
ASV54_17370
ASV54_15105
ASV54_17365
ASV54_17320
ASV54_17325
ASV54_14990
ASV54_15005
ASV54_15010
arsD
ASV54_17335
ASV54_15055
ASV54_17340
ASV54_17345
ASV54_15060
ASV54_15075
ASV54_15270
ASV54_14980
ASV54_14985
ASV54_14995
ASV54_15265
ASV54_15255
ASV54_15240
ASV54_15225
dhaK
dhaL
ASV54_15200
ASV54_15205
ASV54_15185
amaP
ASV54_15150
glf
ASV54_17375
ASV54_15115
ASV54_15120
ASV54_17360
pulA
ASV54_15090
ASV54_15085
ASV54_15080
ASV54_15070
ASV54_17350
ASV54_15065
ASV54_15050
ASV54_17330
ASV54_15045
arsD
arsA
ASV54_15025
arsB
ASV54_15015
ASV54_15000
10 000
20 000
30 000
40 000
50 000
60 000

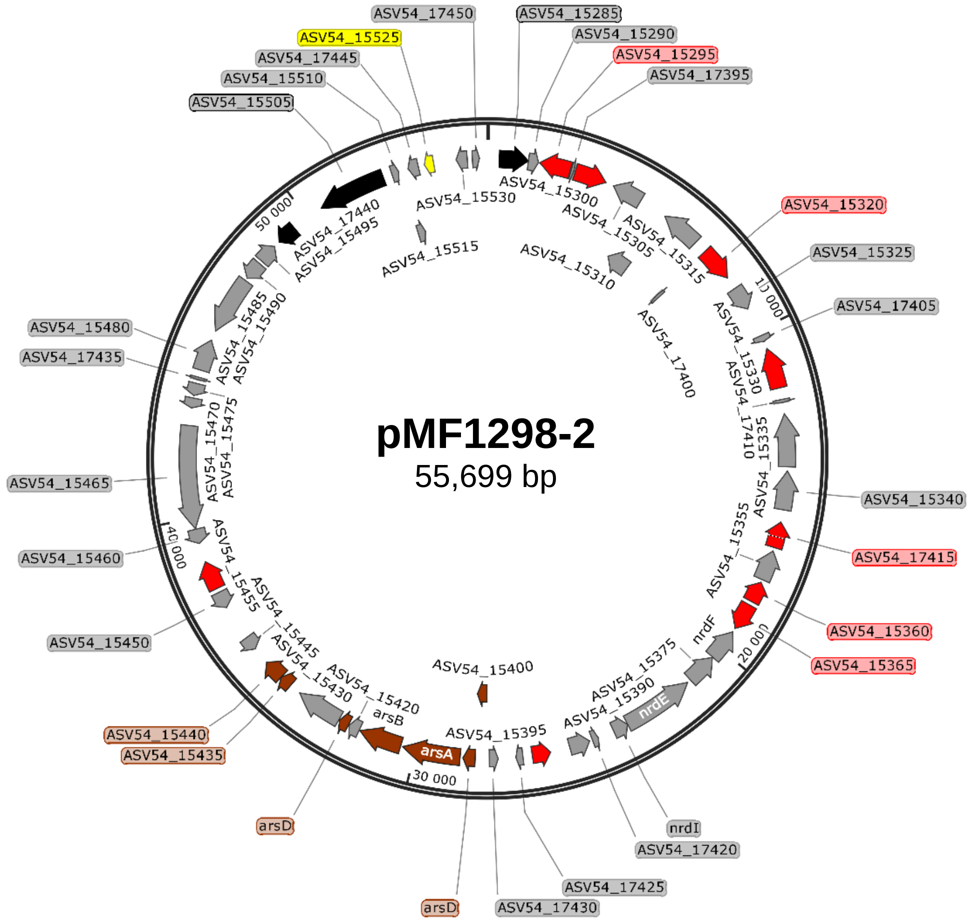
pMF1298-2
55,699 bp
ASV54_17450
ASV54_15525
ASV54_17445
ASV54_15510
ASV54_15505
ASV54_15285
ASV54_15290
ASV54_15295
ASV54_17395
ASV54_15320
ASV54_15325
ASV54_17405
ASV54_15340
ASV54_17415
ASV54_15360
ASV54_15365
nrdI
ASV54_17420
ASV54_17425
ASV54_17430
arsD
arsD
ASV54_15440
ASV54_15435
ASV54_15450
ASV54_15460
ASV54_15465
ASV54_17435
ASV54_15480
ASV54_15530
ASV54_15515
ASV54_17440
ASV54_15495
ASV54_15300
ASV54_15305
ASV54_15310
ASV54_15315
ASV54_17400
ASV54_15330
ASV54_17410
ASV54_15335
ASV54_15355
nrdF
nrdE
ASV54_15375
ASV54_15390
ASV54_15400
ASV54_15395
arsA
arsB
ASV54_15420
ASV54_15430
ASV54_15445
ASV54_15455
ASV54_15470
ASV54_15475
ASV54_15485
ASV54_15490
10 000
20 000
30 000
40 000
50 000

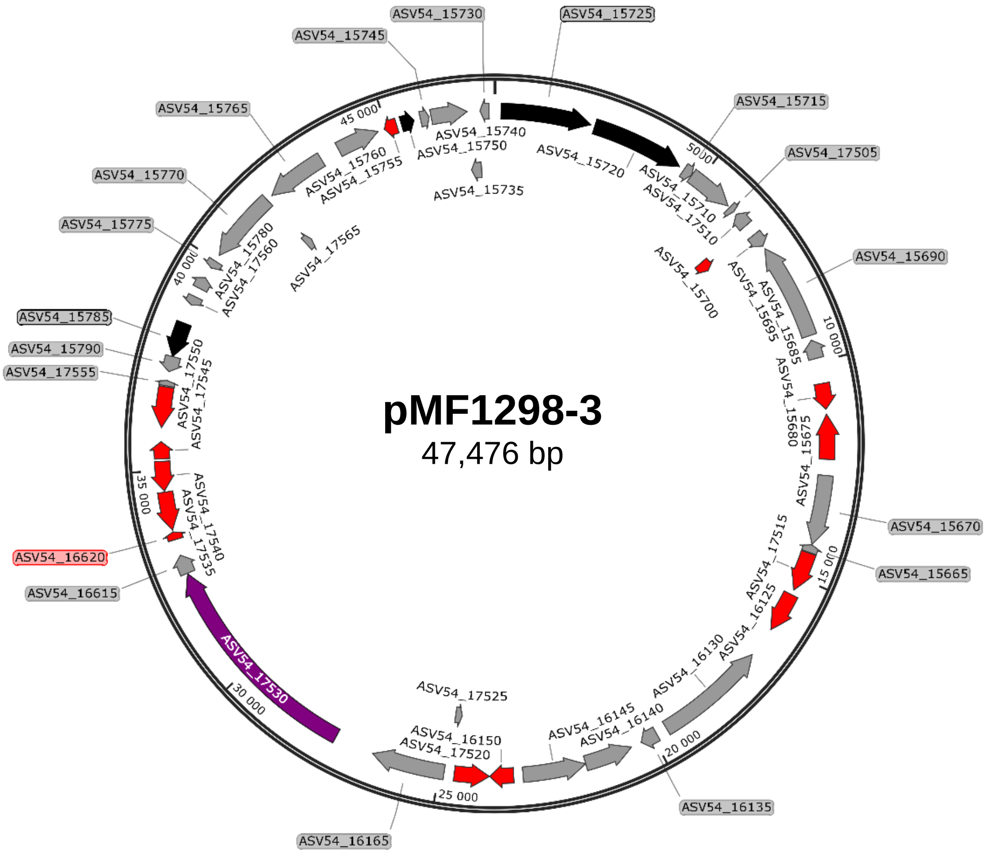
pMF1298-3
47,476 bp
ASV54_15730
ASV54_15725
ASV54_15745
ASV54_15765
ASV54_15715
ASV54_17505
ASV54_15770
ASV54_15775
ASV54_15690
ASV54_15785
ASV54_15790
ASV54_17555
ASV54_15670
ASV54_15665
ASV54_16620
ASV54_16615
ASV54_16135
ASV54_16165
ASV54_15740
ASV54_15750
ASV54_15735
ASV54_15720
ASV54_15710
ASV54_17510
ASV54_15700
ASV54_15760
ASV54_15755
ASV54_15780
ASV54_17560
ASV54_17565
ASV54_15695
ASV54_15685
ASV54_15680
ASV54_15675
ASV54_17515
ASV54_16125
ASV54_16130
ASV54_16145
ASV54_16140
ASV54_17525
ASV54_16150
ASV54_17520
ASV54_17530
ASV54_17550
ASV54_17545
ASV54_17540
ASV54_17535
5000
10 000
15 000
20 000
25 000
30 000
35 000
40 000
45 000

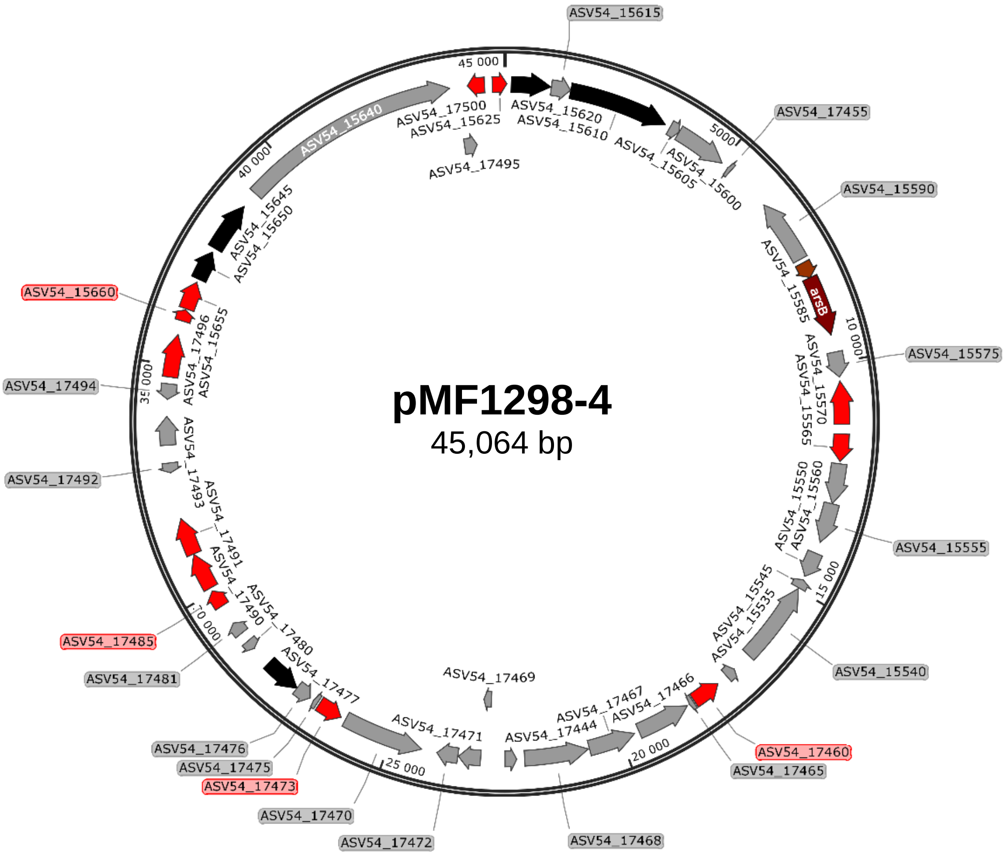
pMF1298-4
45,064 bp
ASV54_15615
ASV54_17455
ASV54_15590
ASV54_15660
ASV54_15575
ASV54_17494
ASV54_17492
ASV54_15555
ASV54_17485
ASV54_15540
ASV54_17481
ASV54_17460
ASV54_17465
ASV54_17476
ASV54_17475
ASV54_17473
ASV54_17470
ASV54_17472
ASV54_17468
ASV54_15640
ASV54_17500
ASV54_15625
ASV54_17495
ASV54_15620
ASV54_15610
ASV54_15605
ASV54_15600
ASV54_15585
arsB
ASV54_15570
ASV54_15565
ASV54_15560
ASV54_15550
ASV54_15545
ASV54_15535
ASV54_15645
ASV54_15650
ASV54_17496
ASV54_15655
ASV54_17493
ASV54_17491
ASV54_17490
ASV54_17480
ASV54_17477
ASV54_17469
ASV54_17471
ASV54_17444
ASV54_17467
ASV54_17466
5000
10 000
15 000
20 000
25 000
30 000
35 000
40 000
45 000

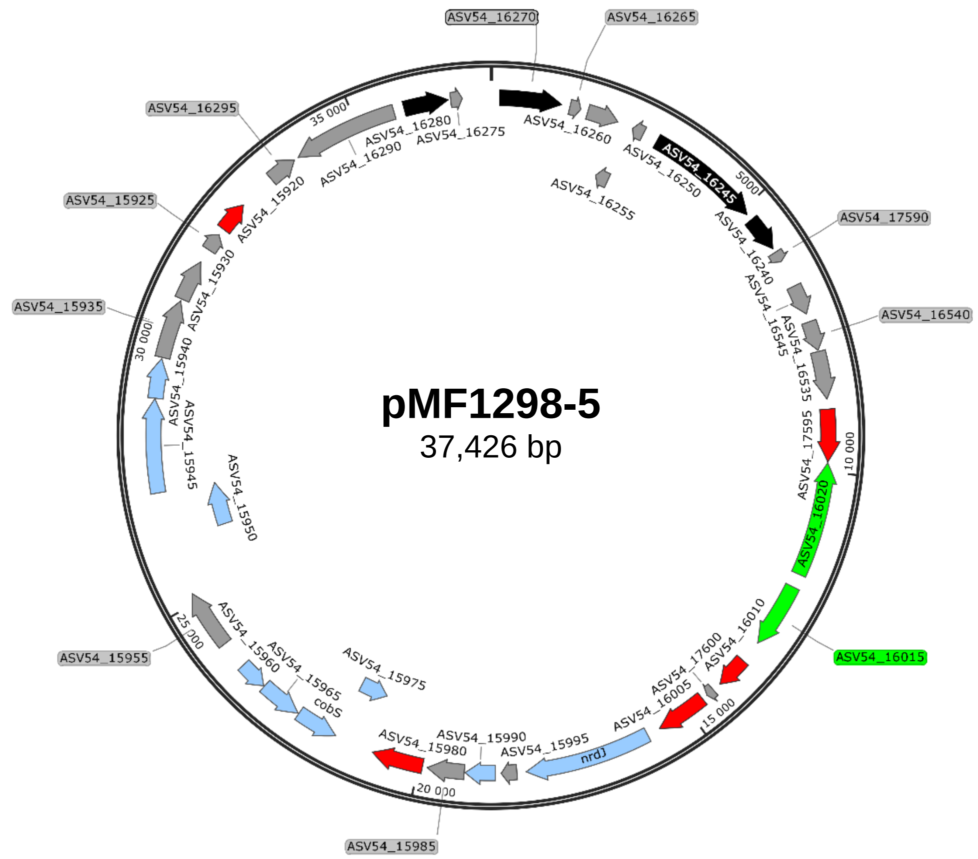
pMF1298-5
37,426 bp
ASV54_16270
ASV54_16265
ASV54_16295
ASV54_15925
ASV54_15935
ASV54_17590
ASV54_16540
ASV54_16015
ASV54_15955
ASV54_15985
ASV54_16280
ASV54_16290
ASV54_16275
ASV54_16260
ASV54_16250
ASV54_16245
ASV54_16255
ASV54_16240
ASV54_16545
ASV54_16535
ASV54_17595
ASV54_16020
ASV54_16010
ASV54_17600
ASV54_16005
ASV54_15995
nrdJ
ASV54_15990
ASV54_15980
ASV54_15975
ASV54_15965
cobS
ASV54_15960
ASV54_15950
ASV54_15945
ASV54_15940
ASV54_15930
ASV54_15920
5000
10 000
15 000
20 000
25 000
30 000
35 000

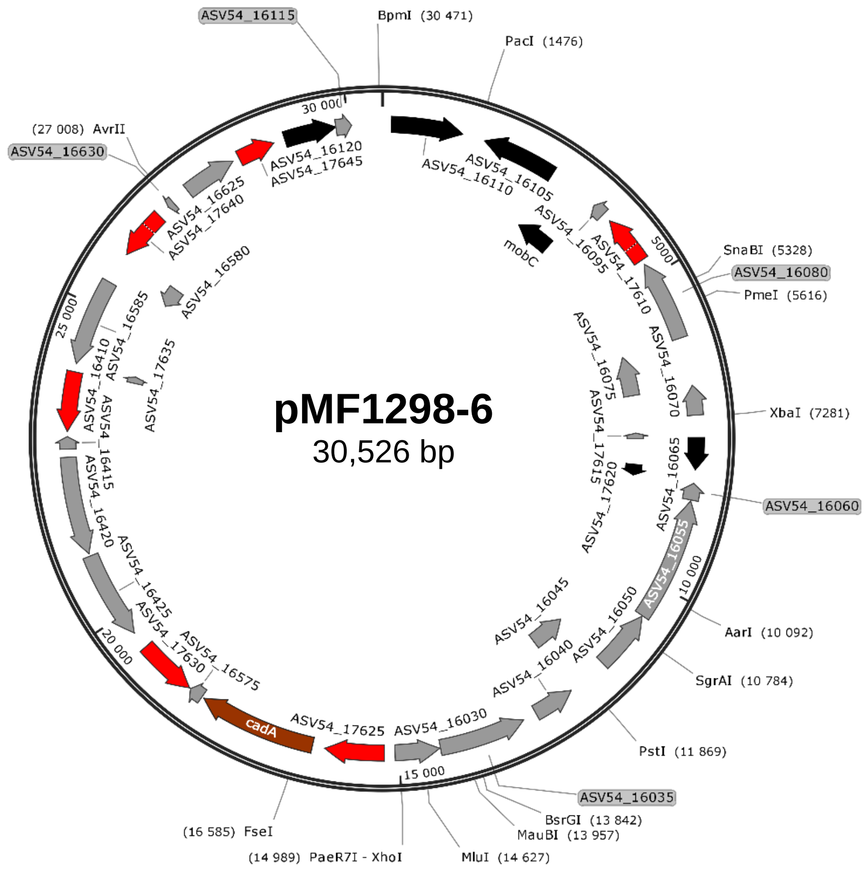
pMF1298-6
30,526 bp
ASV54_16115
BpmI (30 471)
PacI (1476)
(27 008) AvrII
ASV54_16630
SnaBI (5328)
ASV54_16080
PmeI (5616)
XbaI (7281)
ASV54_16060
AarI (10 092)
SgrAI (10 784)
PstI (11 869)
ASV54_16035
BsrGI (13 842)
MauBI (13 957)
MluI (14 627)
(14 989) PaeR7I - XhoI
(16 585) FseI
ASV54_16120
ASV54_17645
ASV54_16110
ASV54_16105
ASV54_16095
mobC
ASV54_17610
ASV54_16070
ASV54_16075
ASV54_17615
ASV54_17620
ASV54_16065
ASV54_16055
ASV54_16050
ASV54_16045
ASV54_16040
ASV54_16030
ASV54_17625
cadA
ASV54_16575
ASV54_17630
ASV54_16425
ASV54_16420
ASV54_16415
ASV54_16410
ASV54_17635
ASV54_16585
ASV54_16580
ASV54_16625
ASV54_17640
5000
10 000
15 000
20 000
25 000
30 000

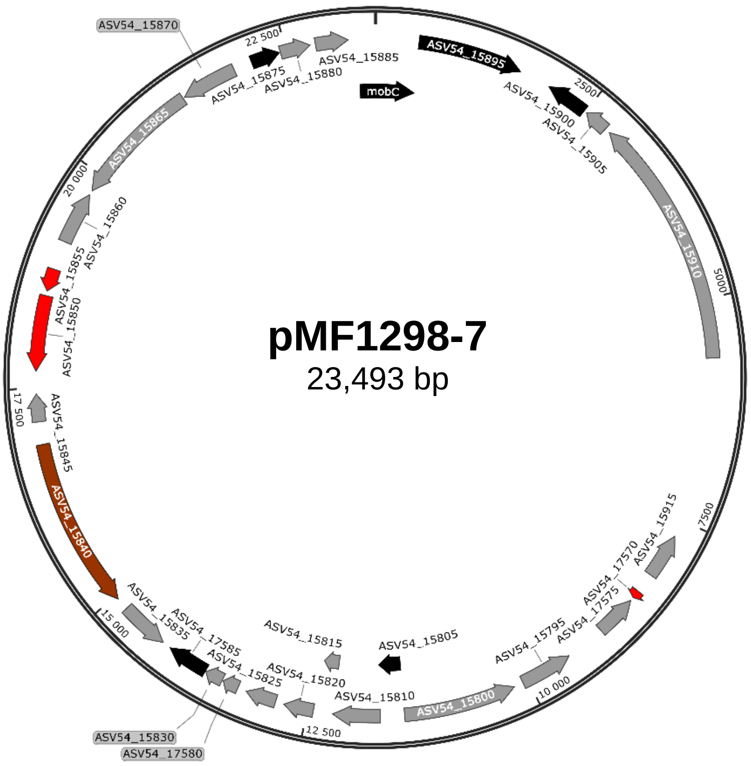
pMF1298-7
23,493 bp
ASV54_15870
22 500
ASV54_15875
ASV54_15880
ASV54_15885
mobC
ASV54_15895
2500
ASV54_15900
ASV54_15905
ASV54_15910
5000
ASV54_15865
20 000
ASV54_15860
ASV54_15855
ASV54_15850
17 500
ASV54_15845
ASV54_15840
7500
ASV54_15915
ASV54_17570
ASV54_17575
ASV54_15795
10 000
ASV54_15800
ASV54_15805
ASV54_15810
ASV54_15815
ASV54_15820
ASV54_15825
ASV54_17585
ASV54_15835
15 000
12 500
ASV54_15830
ASV54_17580

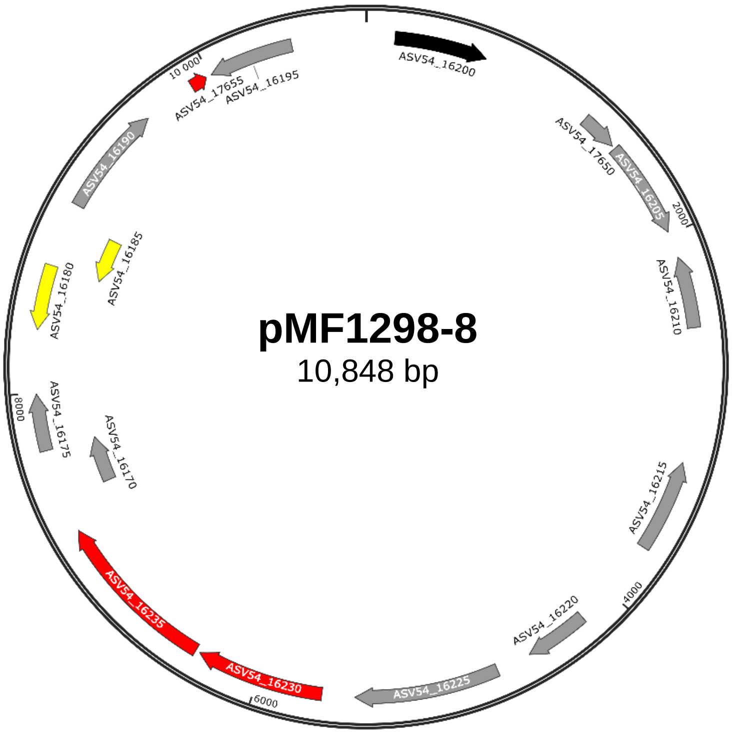
pMF1298-8
10,848 bp
ASV54_16200
10 000
ASV54_17655
ASV54_16195
ASV54_16190
ASV54_17650
ASV54_16205
2000
ASV54_16210
ASV54_16185
ASV54_16180
8000
ASV54_16175
ASV54_16170
ASV54_16215
4000
ASV54_16220
ASV54_16225
ASV54_16230
ASV54_16235
6000

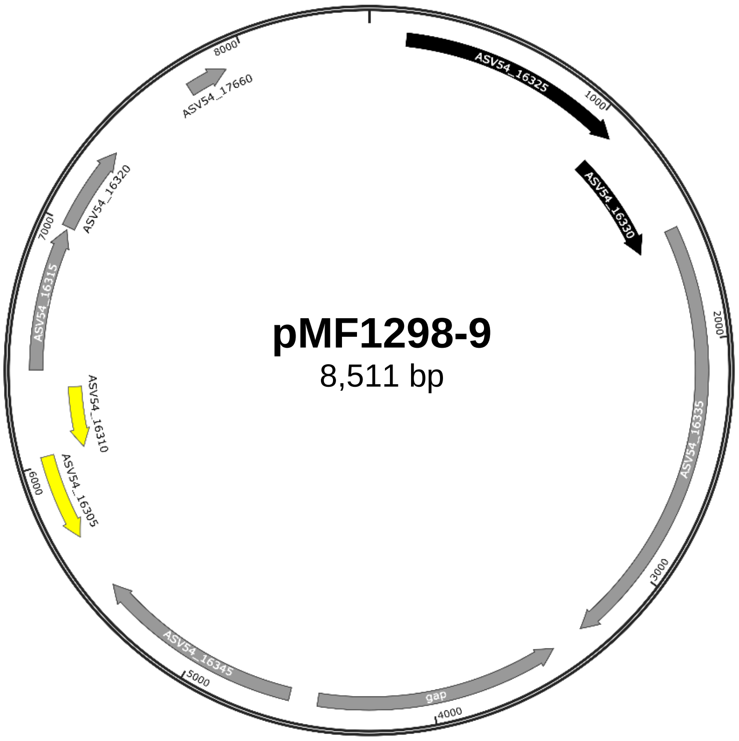
pMF1298-9
8,511 bp
ASV54_16325
ASV54_16330
ASV54_16335
gap
ASV54_16345
ASV54_16305
ASV54_16310
ASV54_16315
ASV54_16320
ASV54_17660
1000
2000
3000
4000
5000
6000
7000
8000

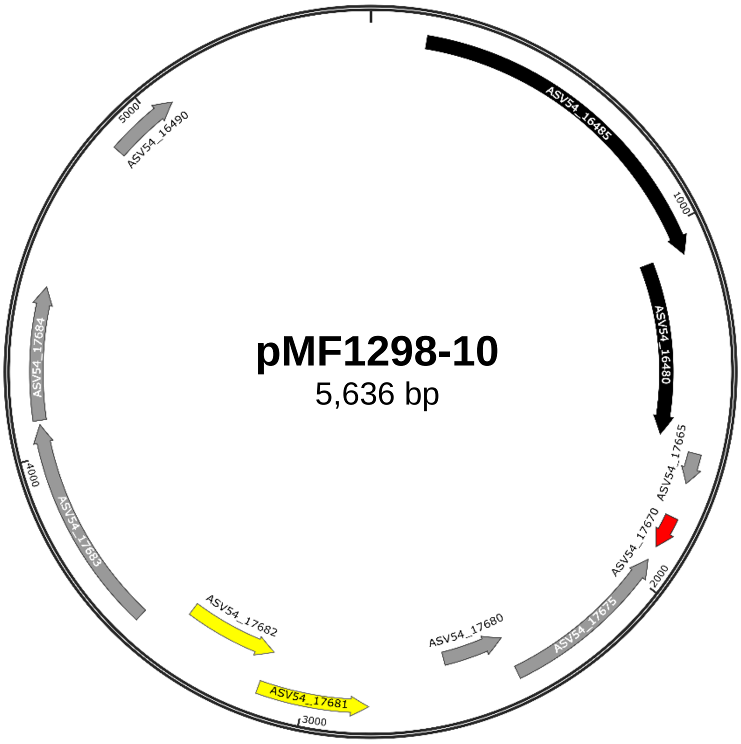
pMF1298-10
5,636 bp
ASV54_16485
ASV54_16480
ASV54_17665
ASV54_17670
ASV54_17675
ASV54_17680
ASV54_17681
ASV54_17682
ASV54_17683
ASV54_17684
ASV54_16490
1000
2000
3000
4000
5000

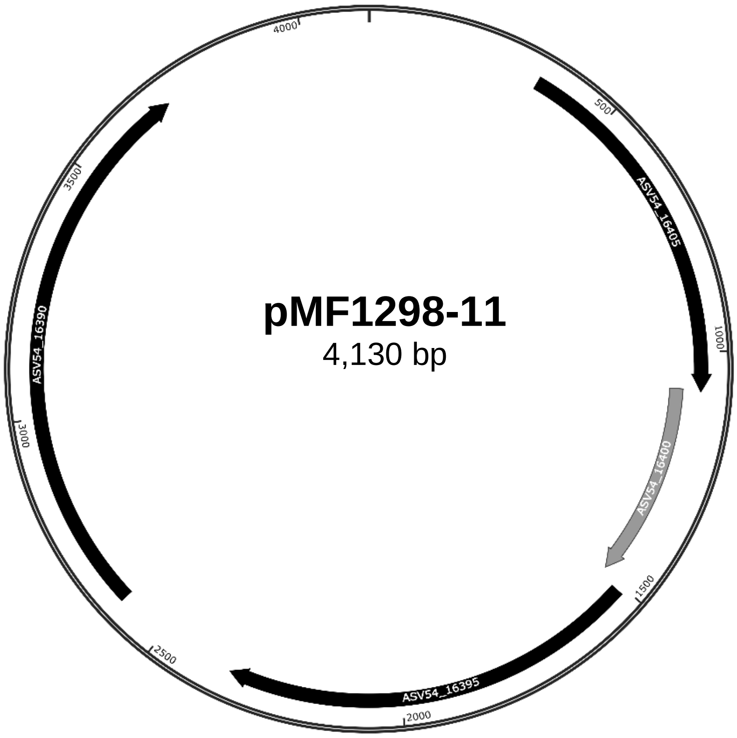
pMF1298-11
4,130 bp
ASV54_16405
ASV54_16400
ASV54_16395
ASV54_16390
500
1000
1500
2000
2500
3000
3500
4000

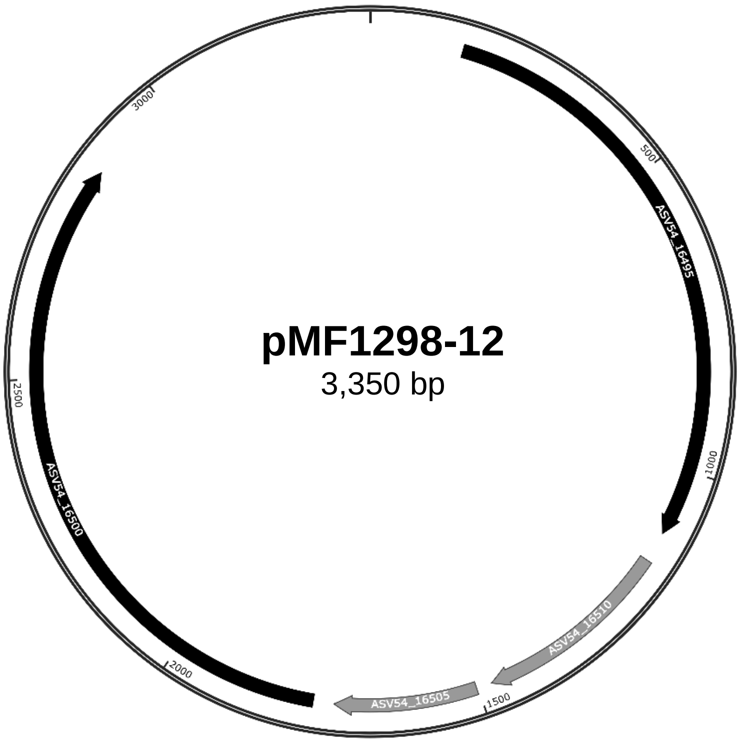
pMF1298-12
3,350 bp
ASV54_16495
ASV54_16510
ASV54_16505
ASV54_16500
500
1000
1500
2000
2500
3000

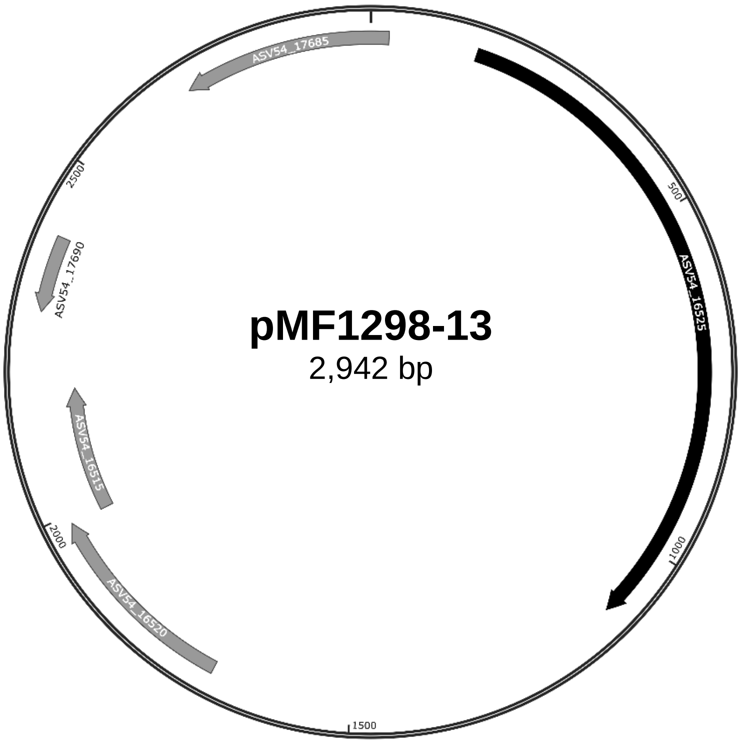
pMF1298-13
2,942 bp
ASV54_17685
ASV54_16525
ASV54_17690
ASV54_16515
ASV54_16520
500
1000
1500
2000
2500

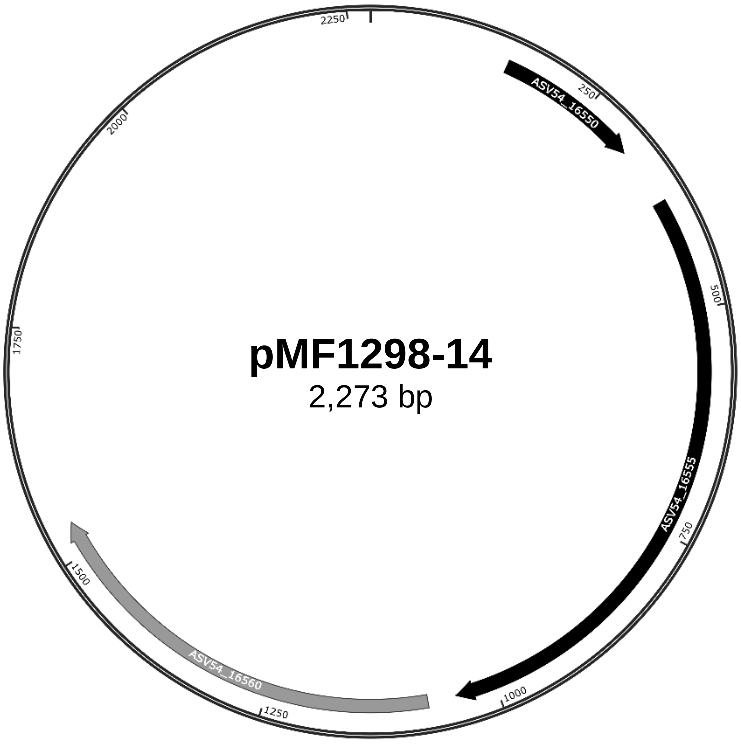
pMF1298-14
2,273 bp
ASV54_16550
ASV54_16555
ASV54_16560
250
500
750
1000
1250
1500
1750
2000
2250
