# Supplementary material for: Large Plasmid Complement Resolved: Complete Genome Sequencing of Lactobacillus plantarum MF1298, a Candidate Probiotic Strain Associated with Unfavorable Effect
Source: Microorganisms. 2019 Aug 14;7(8):262. doi: 10.3390/microorganisms7080262 (PMC6722938; doi:10.3390/microorganisms7080262)
Supplement: Supplementary file 1 [file microorganisms-07-00262-s001.zip › McLeod MF1298_Genome_revised_final_Table S1.pdf]

TABLE S1 | Sequence coverage.

GenBank Assembly[1]	GenBank No.	Type	Name	Size (bp)	Average coverage			
					PacBio	Illumina-T[2]	Illumina-P[2]	Nanopore
GCA_001880185.1 and GCA_001880185.2	CP013149.1	Chromosome	MF1298	3 235 952	80x	1400x[3]	-	-
	CP013150.1	Plasmid	pMF1298-1	63 114	50x	1100x[3]	-	-
	CP013151.1	Plasmid	pMF1298-2	55 699	50x	1100x[3]	-	-
GCA_001880185.2	CP013153.2	Plasmid	pMF1298-3	47 476	-	950x[3]	443x	729x
	CP013152.2	Plasmid	pMF1298-4	45 064	-	900x[3]	379x	683x
	CP013155.2	Plasmid	pMF1298-5	37 426	-	1 200x[3]	504x	855x
	CP013156.2	Plasmid	pMF1298-6	30 526	-	1 350x[3]	475x	475x
	CP013154.2	Plasmid	pMF1298-7	23 493	-	1 750x[3]	645x	372x
	CP013158.2	Plasmid	pMF1298-8	10 848	-	3 650x[3]	1 784x	179x
	CP013160.2	Plasmid	pMF1298-9	8 511	-	4 300x[3]	2 727x	104x
	CP013166.2	Plasmid	pMF1298-10	5 636	-	6 050x[3]	3 871x	66x
	CP013162.2	Plasmid	pMF1298-11	4 130	-	12 050x[3]	12 492x	109x
	CP013167.2	Plasmid	pMF1298-12	3 350	-	10 000x[3]	13 725x	51x
	CP013168.2	Plasmid	pMF1298-13	2 942	-	16 300x[3]	15 979x	80x
	CP013170.2	Plasmid	pMF1298-14	2 273	-	12 200x[3]	14 616x	48x

[1]The initial version 1 (GCA_001880185.1) of the genome assembly was based on PacBio and Illumina sequencing data of total genomic DNA and consisted of 29 entities. It included three circular units, one large chromosome and two plasmids, as well as 26 linear contigs (the linear contigs are not indicated in this table). The three circular units remain unchanged to the second version (Version 2; GCA_001880185.2), consisting of 15 entities, included MinION and Illumina sequencing data of a plasmid DNA enriched fraction which facilitated recovery of complete plasmid structures, and thus in total 14 plasmids were resolved.

[2]Note that the Illumina sequencing of total genomic DNA (Illumina-T) is separate from the Illumina sequencing of plasmid pMF1298-3 to 14 (Illumina-P).

[3]Approximate values based on the complete Illumina assembly of the total genomic DNA, mapped to the MF1298 chromosome and resolved plasmid entities.
